# Identifying Areas with Disproportionate Local Health Department Services Relative to Opioid Overdose, HIV and Hepatitis C Diagnosis Rates: A Study of Rural Illinois

**DOI:** 10.3390/ijerph16060989

**Published:** 2019-03-19

**Authors:** Colleen McLuckie, Mai T. Pho, Kaitlin Ellis, Livia Navon, Kelly Walblay, Wiley D. Jenkins, Christofer Rodriguez, Marynia A. Kolak, Yen-Tyng Chen, John A. Schneider, Whitney E. Zahnd

**Affiliations:** 1 Illinois Department of Public Health, Chicago, IL 60603, USA; Colleen.mcluckie@illinois.gov (C.M.); mpho@bsd.uchicago.edu (M.T.P.); Livia.Navon@illinois.gov (L.N.); Kelly.Walblay@illinois.gov (K.W.); 2 School of Public Health, University of Illinois at Chicago, Chicago, IL 60607, USA; 3 Section of Infectious Diseases and Global Health, Department of Medicine, University of Chicago Medicine, Chicago, IL 60637, USA; ychen22@medicine.bsd.uchicago.edu; 4 Pritzker School of Medicine, University of Chicago Medicine, Chicago, IL 60637, USA; Kaitlin.Ellis@uchospitals.edu; 5 Division of State and Local Readiness, Center for Preparedness and Response, Centers for Disease Control and Prevention, Atlanta, GA 30329, USA; 6 Department of Population Science and Policy, Southern Illinois University School of Medicine, Springfield, IL 62794, USA; wjenkins@siumed.edu (W.D.J.); crodriguez38@siumed.edu (C.R.); 7 Center for Spatial Data Science, University of Chicago, Chicago, IL 60637, USA; mkolak@uchicago.edu; 8 Department of Public Health, Department of Medicine, University of Chicago, Chicago, IL 60637, USA; jschnei1@medicine.bsd.uchicago.edu; 9 Rural and Minority Health Research Center, Arnold School of Public Health, University of South Carolina, Columbia, SC 29208, USA

**Keywords:** opioid use disorder (OUD), persons who inject drugs (PWID), human immunodeficiency virus (HIV), hepatitis C virus (HCV), resource analysis, harm reduction, local health department (LHD), rural health, bivariate mapping, geographic information system (GIS)

## Abstract

*Background:* U.S. rural populations have been disproportionately affected by the syndemic of opioid-use disorder (OUD) and the associated increase in overdoses and risk of hepatitis C virus (HCV) and human immunodeficiency virus (HIV) transmission. Local health departments (LHDs) can play a critical role in the response to this syndemic. We utilized two geospatial approaches to identify areas of discordance between LHD service availability and disease burden to inform service prioritization in rural settings. *Methods:* We surveyed rural Illinois LHDs to assess their OUD-related services, and calculated county-level opioid overdose, HIV, and hepatitis C diagnosis rates. Bivariate choropleth maps were created to display LHD service provision relative to disease burden in rural Illinois counties. *Results:* Most rural LHDs provided limited OUD-related services, although many LHDs provided HIV and HCV testing. Bivariate mapping showed rural counties with limited OUD treatment and HIV services and with corresponding higher outcome/disease rates to be dispersed throughout Illinois. Additionally, rural counties with limited LHD-offered hepatitis C services and high hepatitis C diagnosis rates were geographically concentrated in southern Illinois. *Conclusions:* Bivariate mapping can enable geographic targeting of resources to address the opioid crisis and related infectious disease by identifying areas with low LHD services relative to high disease burden.

## 1. Introduction

The United States is in the midst of a syndemic of opioid-use disorder (OUD), overdose and injection drug-related hepatitis C virus (HCV) infection incidence, with increased risk of human immunodeficiency virus (HIV) among people who inject drugs (PWID) [1]. Recent studies have found that the U.S. populations most vulnerable to OUD and overdose are rural, with limited availability of related services [2,3,4]. An outbreak of opioid injection-related HIV infections in Scott County, Indiana, is illustrative of this geographic vulnerability. Scott County is a small, rural county (population ~24,000) where, during 2014–2015, 181 new cases of HIV were diagnosed primarily among a network of PWID, most of whom were co-infected with HCV [5].

Both the Centers for Disease Control and Prevention (CDC) and the National Association of County and City Health Officials have provided guidance with recommendations for state and local health departments (LHDs) on how to address this syndemic. These include strengthening surveillance and the provision of prevention and intervention services related to OUD, hepatitis C and HIV [2,6]. Many states, including Illinois, have developed opioid action plans that include a broad set of interventions such as OUD prevention, treatment and overdose reversal [7]. Rural LHDs can play a pivotal role in this response by providing services and appropriate referrals. However, rural LHDs face multiple challenges, including lack of resources for identifying high-risk populations and limited capacity to either provide services directly or link patients to services offered by community-based partners [2,8].

Illinois is one of several U.S. states that experienced a statistically significant increase in drug overdose deaths from 2015 to 2016 (34%) and again from 2016 to 2017 (14.3%) [9]. Illinois has received multiple grants from federal partners, including the Substance Abuse and Mental Health Services Administration and the National Institute on Drug Abuse (NIDA), to characterize and respond to this growing problem. One such grant, the State Opioid Response Grant, requires that grantees utilize “epidemiological data to demonstrate the critical gaps in availability of treatment for OUDs in geographic, demographic, and service level terms” [10]. The NIDA grant focuses on addressing the opioid crisis in rural southern Illinois [11]. Similarly, the CDC has provided funding to identify areas of increased vulnerability to the rapid spread of HIV and HCV infection related to opioid use among PWID, to enhance the quality and timeliness of surveillance data, and to implement preventive interventions to reduce overdose [12,13].

We conducted a needs assessment using data from rural Illinois. Our analysis utilized bivariate choropleth mapping, a geographic information system (GIS) approach that is easily implemented and replicable, to evaluate concurrently and display the disease burden and LHD provision of services. This approach can assist state and LHDs in identifying geographic areas with higher disease burden and limited service provision to prioritize public health services and resources and to address the requirements of federal funding and state-level planning [11].

## 2. Materials and Methods

### 2.1. Overview

A survey of Illinois LHD administrators was conducted to assess both OUD and injection-related infectious disease services currently provided by LHDs. We also used disease surveillance data to describe the burden of opioid overdoses, HIV diagnoses, and hepatitis C diagnoses at the county level. We first performed descriptive analyses of access to services and disease burden individually, and then constructed bivariate choropleth maps to display the relationship between these measures. Counties were the units of analysis as they serve as proxies for policy boundaries and most LHDs in Illinois serve individual counties. The analysis was considered non-human subjects research and so was determined to be exempt from review by the University of Chicago Institutional Review Board.

### 2.2. Local Health Department (LHD) Service Provision Survey

Study data were collected and managed using REDCap electronic data capture tools hosted at the Illinois Department of Public Health (IDPH) [14]. The survey included questions to assess the provision and geographic locations of the following LHD services: medication-assisted treatment (MAT), adult mental health services, adult substance use services, youth mental health services, youth substance use services, syringe exchange, syringe disposal, naloxone administration training, naloxone distribution, hepatitis C testing, HIV testing, and HIV pre-exposure prophylaxis (PrEP) services. The survey was reviewed by two LHD administrators (one urban and one rural) to refine content and format.

The survey was sent to each of the 97 Illinois LHD administrators in April 2018. Follow-up emails were sent to non-responding administrators two weeks following the initial email and again, two weeks after the first follow-up email. We used the IDPH Center for Rural Health criteria to identify rural counties; counties were defined as rural if they were not part of a metropolitan statistical area (MSA) or, if they were part of an MSA and had a population of less than 60,000 [15]. Analysis was limited to the responding rural LHDs.

### 2.3. Survey Analysis

Individual services offered in a county were counted according to the number of geographic locations at which the service was provided. We then grouped services into disease-specific service categories: OUD treatment services; HIV prevention and treatment services; and hepatitis C prevention and treatment services. We assessed the median, mean, and range of the number of services provided by LHDs for each disease-specific service grouping.

### 2.4. Disease Surveillance Data

We calculated crude county-level disease diagnosis rates for the most recent years of data available from IDPH. We used 2016–2017 death certificates and emergency department visit data to identify fatal and nonfatal opioid-related overdoses by county [16,17]. Reported diagnoses of HIV (2010–2017) and hepatitis C (2013–2017) by county were obtained from the Illinois HIV disease registry and the Illinois National Electronic Disease Surveillance System, respectively [18,19]. County-level U.S. Census Bureau annual population estimates were aggregated for each time period of disease data available and used to calculate multi-year disease diagnosis rates (opioid-related overdoses, HIV and hepatitis C diagnoses) [20]. We also calculated empirical Bayes (EB) rates in GeoDa version 1.12.1.131 (University of Chicago, Chicago, IL, USA) using the crude county-level disease diagnosis data. EB rates are smoothed rates that address unstable variances and spurious outliers that may occur with the varying population sizes within counties to generate more stable rates [21,22]. Both the crude and smoothed rates are expressed per 100,000 persons.

### 2.5. Choropleth Mapping

A crude service density measure was calculated for all services and for sub-categories (OUD treatment services, HIV prevention and treatment services, and hepatitis C prevention and treatment services) for each county. We also calculated EB service density rates in GeoDa using these data. This measure was calculated by dividing the number of relevant services within a county by the 2017 county-level population estimates from the U.S. Census Bureau (i.e., the county population estimates at the time the LHD survey was conducted) expressed per 100,000 persons [20]. We developed choropleth maps of all disease rates and disease-specific service groups by tertile using both crude and smoothed rates. A quantile classification (i.e., tertiles) was used because the data were highly skewed. Furthermore, for both the crude and smoothed rates of service density, choropleth maps were classed with the lowest density in the darkest color to indicate the scarcest availability of services with lighter colors, indicating greater availability of services.

We then developed bivariate choropleth maps to show the relationship between provision of LHD services and disease burden by tertile of each variable of interest. The following bivariate choropleth maps were developed for both crude and EB rates: (1) opioid overdose rates and OUD treatment services; (2) HIV diagnosis rates and HIV prevention and treatment services; and (3) hepatitis C diagnosis rates and hepatitis C prevention and treatment services. This approach was chosen to display covariation between the two variables of interest (i.e., provision of services and disease burden) [23].

## 3. Results

**Figure 1:**
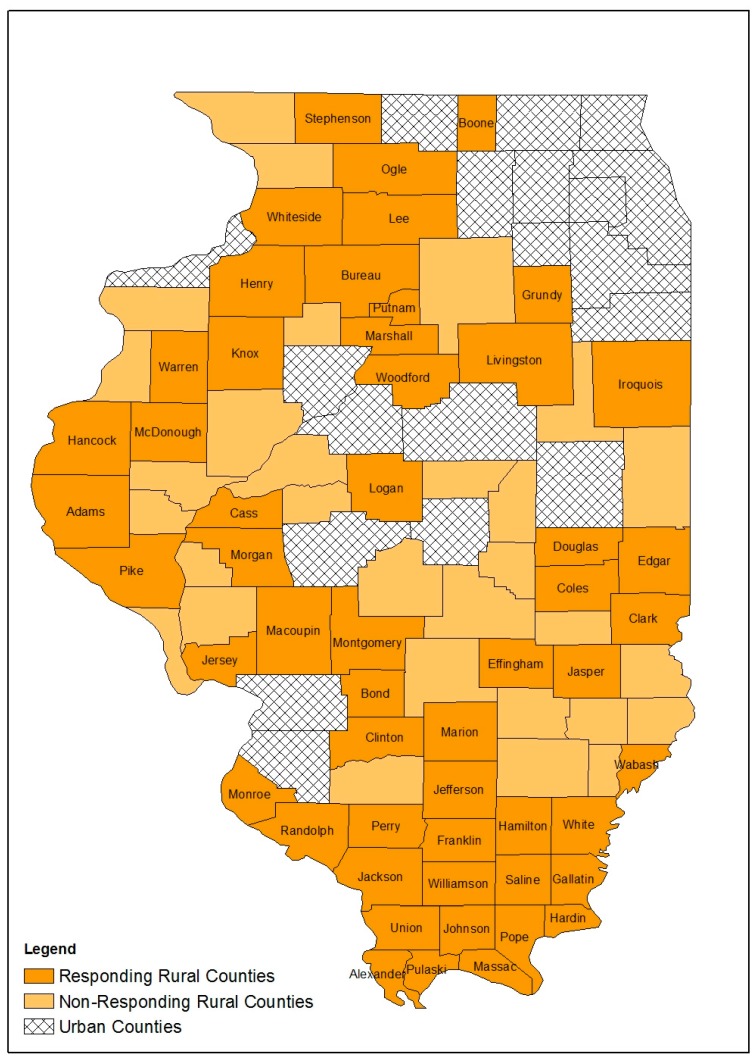
Respondent rural counties, non-respondent rural counties and urban counties, Illinois, 2018.

There are 83 rural counties in Illinois; 53 rural LHDs completed the survey for a response rate of 63.9% (Figure 1). Most responding LHDs offered HIV testing (69.8%) and hepatitis C testing (63.3%) but only 13.2% offered PrEP services (Table 1). Few offered naloxone administration training (28.3%) or naloxone distribution (22.7%); fewer still offered syringe exchange (5.7%) or syringe disposal (15.1%). Similarly, few counties had either youth or adult mental health or substance abuse services available through their LHD, and 86.8 % of LHDs did not offer MAT (Table 1). Responding LHDs offered a median of two HIV-related services and one hepatitis C-related service; the median of OUD treatment services was zero (Table 2).

**Figure 2:**
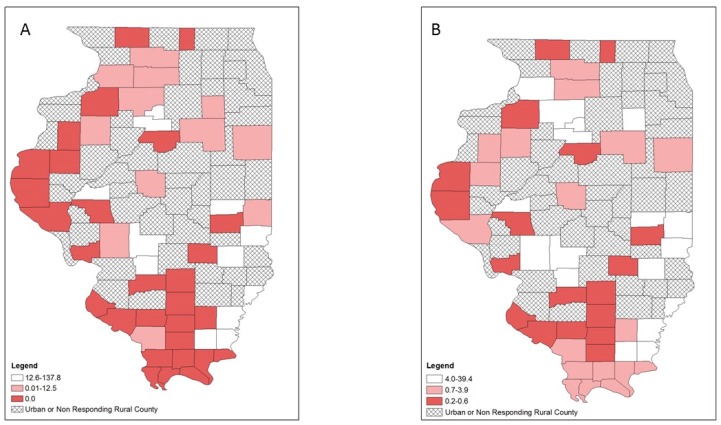
Opioid-use disorder (OUD) treatment services at local health department services per 100,000 population by tertiles, respondent rural counties, Illinois, 2018: (**A**) crude rates; (**B**) empirical Bayes estimated rates.

**Figure 3:**
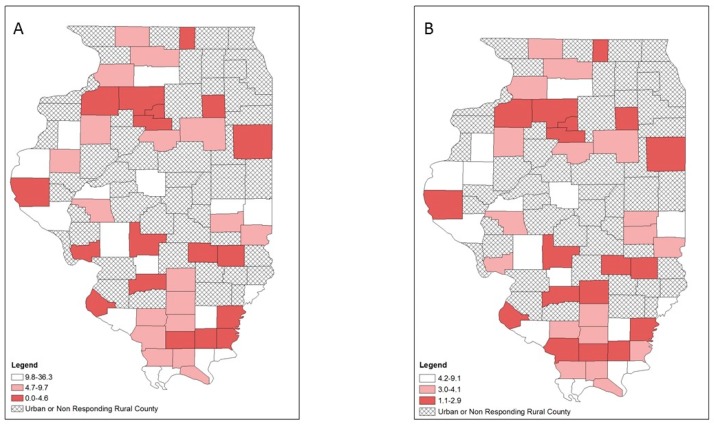
HIV prevention and treatment services at local health department services per 100,000 population by tertiles, respondent rural counties, Illinois, 2018: (**A**) crude rates; (**B**) empirical Bayes estimated rates.

**Figure 4:**
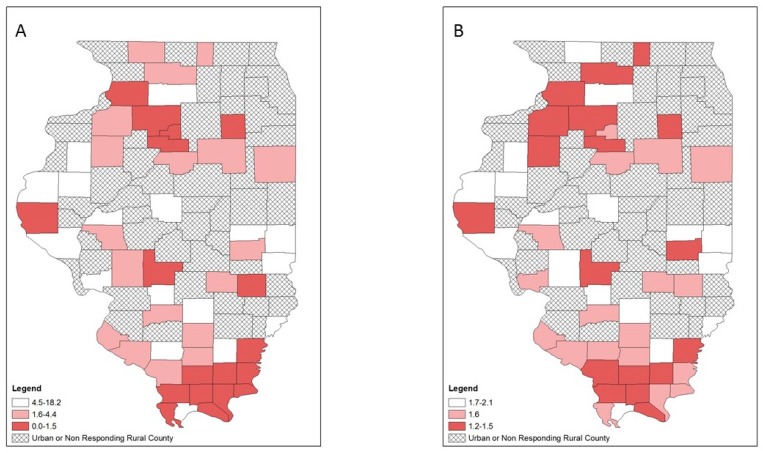
Hepatitis C prevention and treatment services at local health department services per 100,000 population by tertiles, respondent rural counties, Illinois, 2018: (**A**) crude rates; (**B**) empirical Bayes estimated rates.

Lower provision of OUD treatment was primarily located in the western and southern rural counties as indicated by the crude rate map (Figure 2A), while the EB rate map showed a slightly different patterns in which 3 counties in the west and 7 counties in the south were no longer categorized as low OUD treatment provision counties (Figure 2B). For HIV services provision, both the crude rate and EB rate map maps showed that rural counties with low LHD provision of HIV services were scattered in different parts of the state (Figure 3). For hepatitis C services, both the crude rate and the EB rate map showed low LHD provision of hepatitis C services was primarily found in two clusters: northern and southern rural counties (Figure 4). However, several northern and northwestern rural counties further became low hepatitis C service provision counties on the EB rate map, while some southern rural counties were no longer low hepatitis C service provision counties.

**Figure 5:**
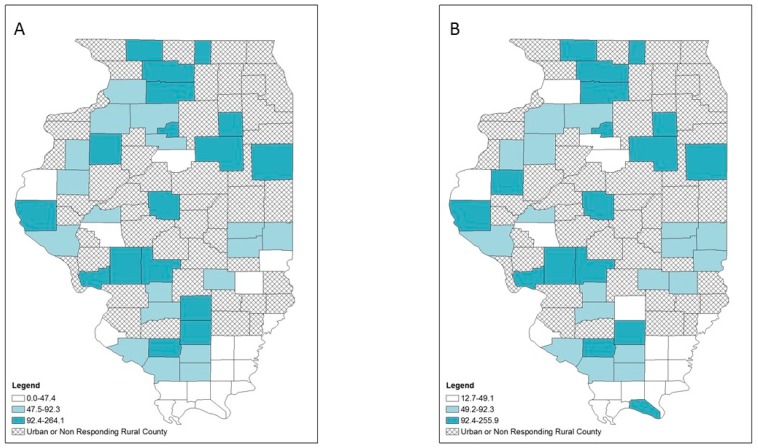
Opioid-related overdose rates per 100,000 population by tertiles, respondent rural counties, Illinois, 2016–2017: (**A**) crude rates; (**B**) empirical Bayes estimated rates.

**Figure 6:**
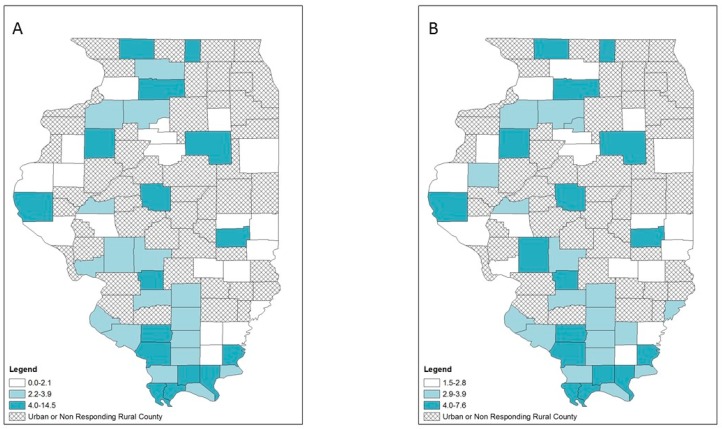
HIV diagnosis rates per 100,000 population by tertiles, respondent rural counties, Illinois, 2010–2017: (**A**) crude rates; (**B**) empirical Bayes estimated rates.

**Figure 7:**
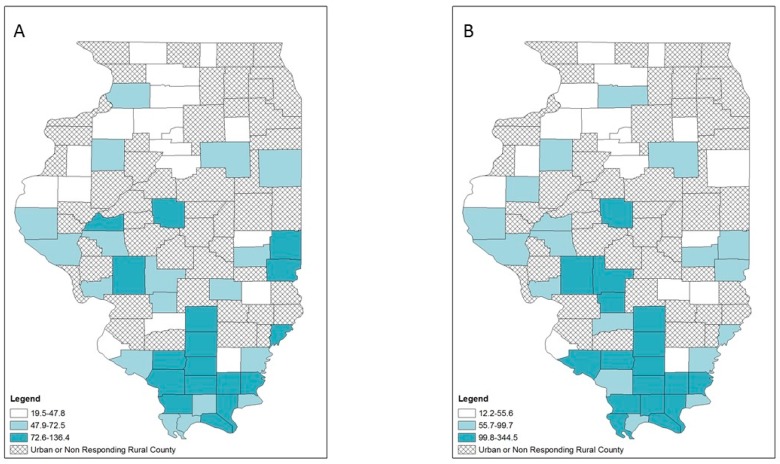
Hepatitis C diagnosis rates per 100,000 population by tertiles, respondent rural counties, Illinois, 2010–2017: (**A**) crude rates; (**B**) empirical Bayes estimated rates.

Opioid overdose, HIV and hepatitis C diagnosis rates also varied geographically (Figure 5, Figure 6 and Figure 7). Counties in the highest tertile of opioid overdose rates tended to be among central and northern rural counties (Figure 5). Compared to the crude rate map (Figure 5A), the EB rate map (Figure 5B) showed one county in the central southern region n the lowest tertile of opioid overdose rate compared to being in the highest tertile of opioid overdose rate. Rural counties in the highest tertile of HIV diagnosis rates occurred across Illinois, with a small cluster in the southernmost counties (Figure 6). For hepatitis C diagnosis rates, the crude rate and EB rate maps (Figure 7A,B) showed that the rural counties in the highest tertile of hepatitis C diagnosis rates were mostly in southern Illinois.

**Figure 8:**
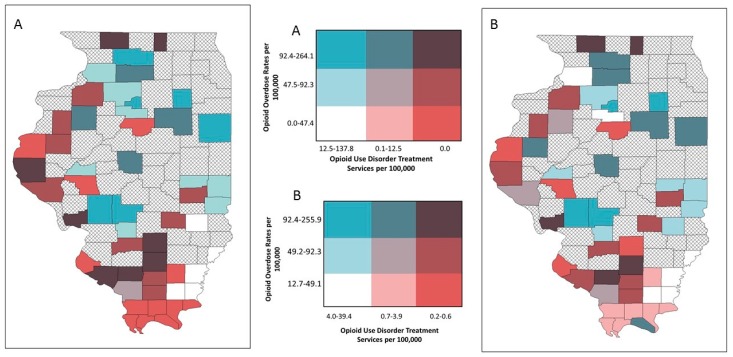
Bivariate choropleth maps of local health department services and disease burden, respondent rural counties, Illinois: opioid-use disorder (OUD) treatment services and opioid overdose rates; (**A**) crude rates (**B**) empirical Bayes estimated rates.

**Figure 9:**
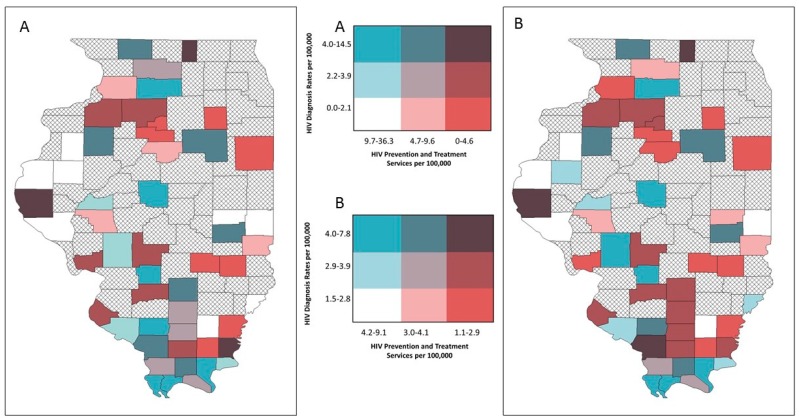
Bivariate choropleth maps of local health department services and disease burden, respondent rural counties, Illinois: HIV prevention and treatment services and HIV diagnosis rates; (**A**) crude rates (**B**) empirical Bayes estimated rates.

**Figure 10:**
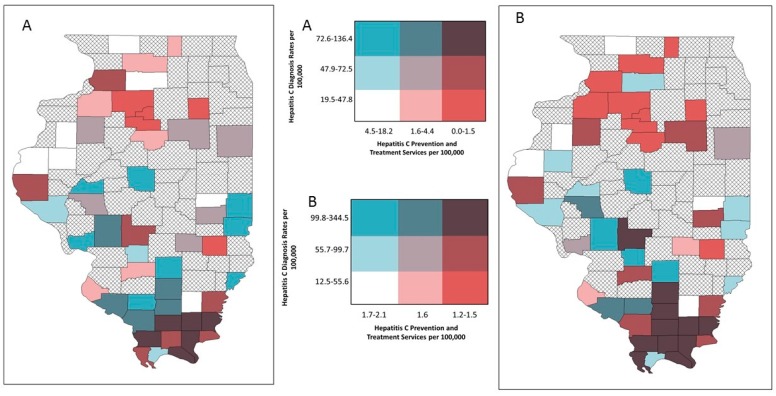
Bivariate choropleth maps of local health department services and disease burden, respondent rural counties, Illinois: hepatitis C prevention and treatment services and hepatitis C diagnosis rates; (**A**) crude rates (**B**) empirical Bayes estimated rates.

Bivariate mapping showed areas of high opioid overdose rates and low LHD provision of OUD treatment resources in different parts of the state (Figure 8). This was similar for HIV diagnosis rates and services (Figure 9). However, several central southern rural counties emerged on the EB rate map that indicated low LHD OUD treatment provision and relatively high HIV diagnosis rates (Figure 9B). For hepatitis C, both the crude rate map and the EB rate map showed that counties with high hepatitis C diagnosis rates and low provision of corresponding services were concentrated in southern Illinois (Figure 10A), and this pattern particularly emerged on the EB rate map (Figure 10B).

## 4. Discussion

We found that most rural counties in Illinois provided few harm reduction services, PrEP clinics, MAT, and naloxone-related services. While counties with low provision of OUD treatment services and HIV services and corresponding high diagnosis rates were more generally dispersed, counties with low LHD provision of hepatitis C services and high rates of hepatitis C diagnosis were primarily located in the southern part of the state. Interestingly, the southernmost 16 counties in Illinois are part of the federally designated Delta Regional Authority, an area of 252 counties and parishes described as the most economically distressed area of the country with low access to health care services like primary care [24,25]. Further research is needed to explore these findings.

Of note, counties of highest discordance between disease burden and LHD service availability were not consistent across all three disease categories, demonstrating that jurisdictions could prioritize specific interventions (i.e., naloxone distribution in areas of opioid overdose discordance vs. syringe services in areas of hepatitis C discordance) to optimize public health impact and strategically utilize available resources. This is critical for rural LHDs, who often face the dual challenge of limited resources and personnel for planning and implementation, as well as the increased responsibility to provide a broad range of programs including direct health services for their communities [8]. Service/need mapping as described in this analysis provides a simple, replicable framework for states to support their local jurisdictions in utilizing limited resources.

Comparing results across both crude and adjusted data, empirical Bayes rates are helpful for identifying persistent trends and assessing the sensitivity of results. Empirical Bayes smoothing stabilizes rates by borrowing strengths from other spatial units of the sample, using global characteristics of the whole study region to define a prior distribution. The assessment of prevention and treatment service rates in particular was sensitive to small reported numbers per county and skewed distribution, making adjusted rates essential to defining true patterns. Adjusted rates additionally made regional trends explicit in bivariate choropleth maps, highlighting persistent areas of relative low access and high disease burden—especially in southern Illinois.

GIS approaches have frequently been used for disease surveillance as well as health policy and planning purposes [26]. Specifically, GIS approaches can supplement commonly utilized health assessment approaches and frameworks, such as the Mobilizing for Action through Planning and Partnerships (MAPP) framework [27]. In Illinois, this approach may help LHDs develop their Illinois Project Local Assessment of Needs (IPLAN), an assessment process based on MAPP that each LHD must complete every five years to prioritize health needs and subsequently address them through the development of objectives, strategies and plans to implement those strategies [28]. Many rural LHDs in Illinois have multiple counties in their jurisdictions. Mapping these data may help them geographically target their efforts in counties of greatest need within their jurisdictions.

Others have noted that simultaneously mapping health services and population need is an effective use of GIS for LHD priority setting and program planning; however, bivariate choropleth mapping has been an underutilized approach for mapping health services and disease burden [29]. Previous studies have overlaid point data of health service locations on a choropleth map of disease burden or have overlaid proportional symbols (e.g., dots of increasing size indicating greater numeric values) noting varying levels of service access over a choropleth map of disease burden. Both approaches display information using two types of symbols [30]. Bivariate maps, on the other hand, display information on service provision and disease burden using a single symbol type, which allow discordant areas to be identified easily.

### Limitations and Strengths

Our study has several limitations. First, we did not receive a response from all rural LHDs in the state, giving us an incomplete representation of the provision of services by rural LHDs. Second, we assessed solely services provided by LHDs, not those provided by 87 rural federally qualified health centers, 233 rural health clinics, and other primary care providers, hospitals, and non-profit organizations; therefore, OUD-related resources available to rural populations may be underestimated [31]. For example, MAT can be provided by health centers through Drug Abuse Treatment Act (DATA) waivers [32]. Additionally, some LHDs may provide services within a single county but make those services available for multiple counties; evaluating “service areas” of available LHD services may be an important area for future research. The disease surveillance data utilized in this analysis may be incomplete due to undiagnosed or underreported infection or overdose misdiagnosis. Also, while our survey did elicit the names and locations of active LHD services, it did not provide information on current service volumes or existing capacity. Furthermore, we express all service densities and diagnosis rates per 100,000 persons. Container measures such as these are limiting for assessing service density as they assume that people only access services within their own county. However, we use this expression of rates because it is commonly used in the presentation of surveillance data like the disease rates that we present in this paper, and thus may be useful for public health practitioners. Spatial statistical testing, such as Moran’s I or Geary C, was not performed, in part due to analysis being limited to only rural Illinois counties (i.e., spatial discontinuity). Finally, while bivariate mapping is easy to implement and can effectively display two variables simultaneously, more research is needed to determine how utilization of these maps can influence resource allocation and planning [23,33].

Our study’s strengths include the replicability of the survey development, dissemination, and analysis, particularly the crude rate analysis. Additionally, our framework allows for quick and accessible needs assessments that can accommodate limited resources. Our analysis is unique in its attempt to gather health department service availability at the local jurisdictional level, and to overlay this information with disease surveillance data. Furthermore, in addition to crude rates, we calculated EB estimated rates to help reduce problematic variances and outliers.

## 5. Conclusions

Our analysis proposes a framework for states and LHDs to perform rapid needs assessments of OUD, overdose, and related HIV diagnosis and hepatitis C diagnosis rates using simple survey and GIS methods. Such approaches may help to prioritize resource allocation in rural areas with low levels of opioid-related services, and higher rates of opioid overdoses and related infectious diseases.

## Figures and Tables

**Table 1:** Number of opioid use disorder (OUD), hepatitis C and human immunodeficiency virus (HIV) service locations reported by rural local health departments, Illinois, 2018.

Service	N (%)	Disease-Specific Service Group
HIV testing		HIV prevention and treatment services
0	16 (30.2%)	
1	33 (62.3%)	
2+	4 (7.5%)	
Hepatitis C testing		Hepatitis C prevention and treatment services
0	20 (37.7%)	
1	29 (54.7%)	
2+	4 (7.6%)	
Pre-exposure Prophylaxis (PrEP) Clinic		HIV prevention and treatment services
0	46 (86.8%)	
1	6 (11.3%)	
2+	1 (1.9%)	
Naloxone Administration		OUD treatment services
0	38 (71.7%)	
1	13 (24.5%)	
2+	2 (3.8%)	
Naloxone Distribution		OUD treatment services
0	41 (77.4%)	
1	11 (20.8%)	
2+	1 (1.9%)	
Syringe Exchange		HIV & hepatitis C prevention and treatment services
0	50 (94.3%)	
1	3 (5.7%)	
2+	0 (0%)	
Syringe Disposal		HIV & hepatitis C prevention and treatment services
0	45 (84.9%)	
1	8 (15.1%)	
2+	0 (0%)	
Adult Mental Health		OUD treatment services
0	37 (69.8%)	
1	13 (24.5%)	
2+	3 (5.7%)	
Youth Mental Health		OUD treatment services
0	38 (71.7%)	
1	12 (22.6%)	
2+	3 (5.7%)	
Adult Substance Use		OUD treatment services
0	41 (77.4%)	
1	10 (18.9%)	
2+	2 (3.8%)	
Youth Substance Use		OUD treatment services
0	41 (77.4%)	
1	10 (18.9%)	
2+	2 (3.8%)	
Medication-Assisted Therapy		OUD treatment services
0	48 (86.8%)	
1	6 (11.3%)	
2+	1 (1.9%)	

**Table 2:** Number of services by disease-specific service group reported by rural local health departments, Illinois, 2018.

Disease-Specific Service Group	Mean (SD)	Median (Range)
Opioid Use Disorder (OUD) treatment services	1.9 (2.8)	0 (0–13)
HIV prevention and treatment services	2.0 (1.6)	2 (0–9)
Hepatitis C prevention and treatment services	0.9 (0.8)	1 (0–3)
